# Outcomes of Unrestricted Weight-Bearing During Interval Period With Cement-on-Cement Articulating Antibiotic-Loaded Spacers in Two-Stage Revision for Knee Prosthetic Joint Infection

**DOI:** 10.7759/cureus.75404

**Published:** 2024-12-09

**Authors:** Kazuya Uehara, Eiichi Shiigi, Kazushige Seki, Takashi Imagama, Toshihiro Seki, Hiroshi Tanaka, Takashi Sakai

**Affiliations:** 1 Department of Orthopedic Surgery, Yamaguchi University Graduate School of Medicine, Ube, JPN; 2 Department of Orthopedic Surgery, Yamaguchi Prefectural Grand Medical Center, Hofu, JPN

**Keywords:** articulating spacer, full weight-bearing, prosthetic joint infection, total knee arthroplasty, two-stage revision

## Abstract

Background: Two-stage revision is known as the gold-standard method for knee prosthetic joint infection (PJI), but the most suitable treatment method remains controversial. Typically, weight-bearing is restricted during the interval between the stages. The aim of this study was to evaluate the clinical outcomes of unrestricted weight bearing with cement spacers fabricated using the Knee Articulating Spacer Mold (KASM®; Ortho Development Corporation, Draper, UT, USA) for knee PJI.

Methods: We retrospectively reviewed 16 patients who underwent two-stage revision surgery for knee PJI between April 2015 and March 2020. The procedure involved the removal of the infected prosthetic joints and the insertion of cement spacers made using KASM®. The evaluation focused on the possibility of full-weight bearing gait during the interval between the first and second stages, surgical time, blood loss, complications, and postoperative outcomes, including the Knee Society Score (KSS), Knee Society Function Score, and range of motion (ROM).

Results: All patients were able to walk with full weight-bearing. However, cement spacer dislocation occurred in one patient (6.3%). During the interval between stages, infection occurred in one patient (6.3%) and debridement was performed. Average interval between the stages was 92.7 days (range, 55-166 days). After the reimplantation, reinfection occurred in two patients (12.5%) out of the 16. Among the 14 patients with successful reimplantation, the average operative time was 116.1 min (range, 76-153 min) and the average perioperative blood loss was 476.1 mL (range, 89.5-859 mL). The KSS was 86.4 (range, 62-100), the Knee Society Function Score was 73.6 (range, 45-100), and flexion ROM was 111.8° (range, 95°-130°) at the latest follow-up. The mean follow-up period was 871 days (range, 117-1103 days).

Conclusions: Unrestricted weight-bearing gait using cement spacers during the waiting period for two-stage revision surgery for knee PJI led to favorable outcomes in this case series, which lacked a control group. Further studies are needed to assess whether the benefits of weight-bearing outweigh the risks and improve overall outcomes.

## Introduction

Prosthetic joint infection (PJI) is one of the most serious complications of knee arthroplasty, with a reported incidence of 0.5%-2.0% [1]. Although several studies have been conducted, there is still no consensus on the most suitable treatment method.

In the commonly used two-stage revision surgery for knee PJI, a cement spacer is generally implanted in the first stage. To date, there have been several reports on the therapeutic outcomes following two-stage revision using static spacers [2] and articulating spacers including cement-on-cement [3], cement-on-polyethylene [4], and metal-on-polyethylene spacers [5,6]. Articulating spacers confer mobility to the affected joint during the first stage of revision, which is their major advantage. Moderate range of motion (ROM) exercises are effective for maintaining the length and elasticity of the extensor mechanism, which is useful for preventing scarring of the soft tissue surrounding the joint, shortening of the quadriceps muscles, and hardening and contraction of the articular capsule [2,7,8]. However, weight-bearing during the interval period between stages is commonly restricted. This restriction poses a significant challenge, particularly for elderly or mobility-compromised patients, as prolonged weight-bearing restrictions can result in muscle weakness, deconditioning, and loss of independence. The stability is critical in enabling weight-bearing.

Various types of spacers are employed in two-stage revision surgery, including handcrafted spacers [3,9], those produced with metal molds [10], and those created using silicone molds [11,12]. We have used a type of cement-on-cement articulating antibiotic-loaded spacer (Knee Articulating Spacer Molds, KASM®; Ortho Development Corporation, Draper, UT, USA) for two-stage revision procedures since 2015. The KASM® spacer stands out due to its unique design and functional advantages. Unlike most femoral spacers, which typically feature a closed design, the KASM® spacer incorporates an open femoral mold that can be directly compressed onto the femur. This design facilitates the accommodation of irregularities or defects in the femoral bone, ensuring a secure fit and improved stability. The silicone-based tibial mold further enhances the spacer’s adaptability by allowing preoperative or intraoperative adjustments. Spacer thickness can be precisely tailored to match the joint gap, such as the extension or flexion gap, optimizing joint space and soft tissue balance. For patients with significant extension gaps, additional cement can be applied beneath the tibial mold to adjust joint alignment and improve stability. Furthermore, the KASM® includes three sizes for both femoral and tibial components, enabling it to accommodate a wide variety of knee joint anatomies. These features collectively support unrestricted weight-bearing between stages while maintaining ROM, proper joint space, and soft tissue balance.

The present study aimed to elucidate the achieving full weight-bearing gait and clinical outcomes with implantation of cement spacers constructed by using KASM during the waiting period of two-stage revision surgery for knee PJI.

## Materials and methods

This retrospective study was approved by the local ethics committee, and all analyses were performed after obtaining permission from the Institutional Review Board of the authors' institution (Yamaguchi Prefectural Grand Medical Center, Approval No. 2020-J029). Informed consent was obtained from each patient. We investigated 23 patients who underwent surgery for knee PJI, involving the removal of their prosthetic joints and the insertion of cement spacers created using KASM® as part of a two-stage revision procedure. Data were obtained from the electronic medical records. Five cases with a postoperative follow-up period after reimplantation of less than three months were excluded. Two patients, deemed high-risk for infection due to advanced age and significant medical comorbidities that rendered highly invasive surgery unfavorable, underwent only the first stage of the two-stage revision with cement spacer retention and were excluded from the study. As a result, the final study group consisted of 16 patients. There were 15 total knee arthroplasty (TKA) infections and one unicompartmental knee arthroplasty (UKA) infection. The patients included seven males and nine females with a mean age of 73.1 years (range, 58-94). The mean heights, body weights, and body mass indices (BMI) were 155.5 cm (range, 138-180 cm), 61.5 kg (range, 47.1-90.5 kg), and 25.4 (range, 19.3-34.9), respectively. No patients with bilateral infections were included in the present study. Considering comorbidities, one patient had diabetes mellitus, and three patients had rheumatoid arthritis (Table 1).

**Table 1:** Patient Demographics and Clinical Characteristics The data represented as mean ± standard deviation BMI: body mass index, TKA: total knee arthroplasty, UKA: unicompartmental knee arthroplasty

Variables	Values
Mean Age (years)	73.1 ± 9.4
Sex	7 males, 9 females
Mean Height (cm)	155.5 ± 10.7
Mean Body Weight (kg)	61.5 ± 12.7
Mean BMI (kg/m^2^)	25.4 ± 4.1
Comorbidities	1 Diabetes mellitus, 3 Rheumatoid arthritis
Type of prothesis	15 TKA, 1 UKA

The duration from the initial knee surgery to diagnosis of PJI was 27.7 months (range, 27 days-105.6 months).

The organisms identified were as follows: methicillin-sensitive Staphylococcus aureus (MSSA) in eight cases, methicillin-resistant Staphylococcus aureus (MRSA) in three cases, coagulase-negative staphylococci (CNS) in two cases, Mycobacterium intracellulare in one case, negative cultures in one case, a combination of CNS and Candida glabrata in one case, and a combination of Staphylococcus capitis and S. epidermidis in one case.

Surgical technique for first-stage of revision surgery (cement spacer insertion)

**Figure 1:**
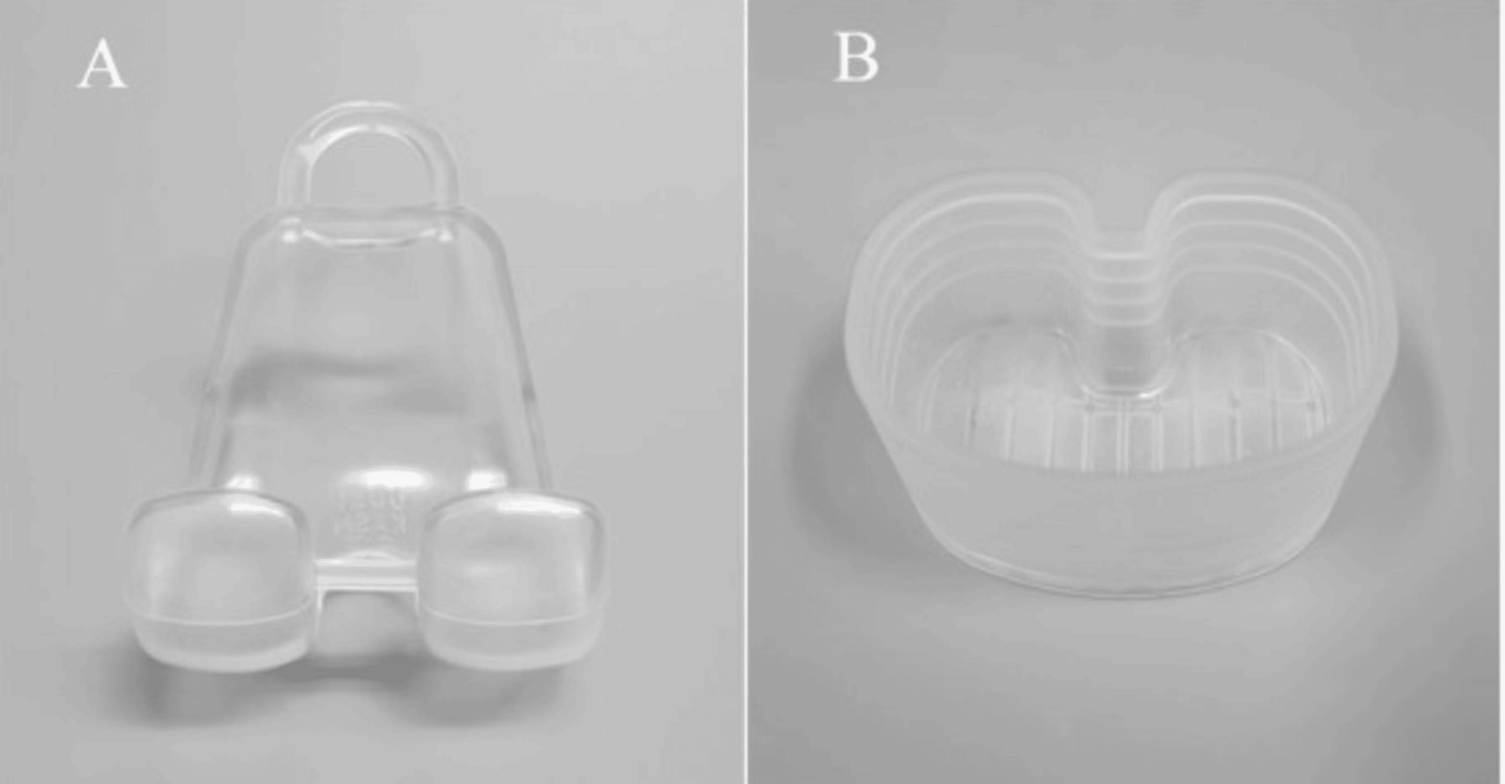
A photograph of a femoral mold (A) and a tibial mold (B) of Knee Articulating Spacer Mold (KASM®).

**Figure 2:**
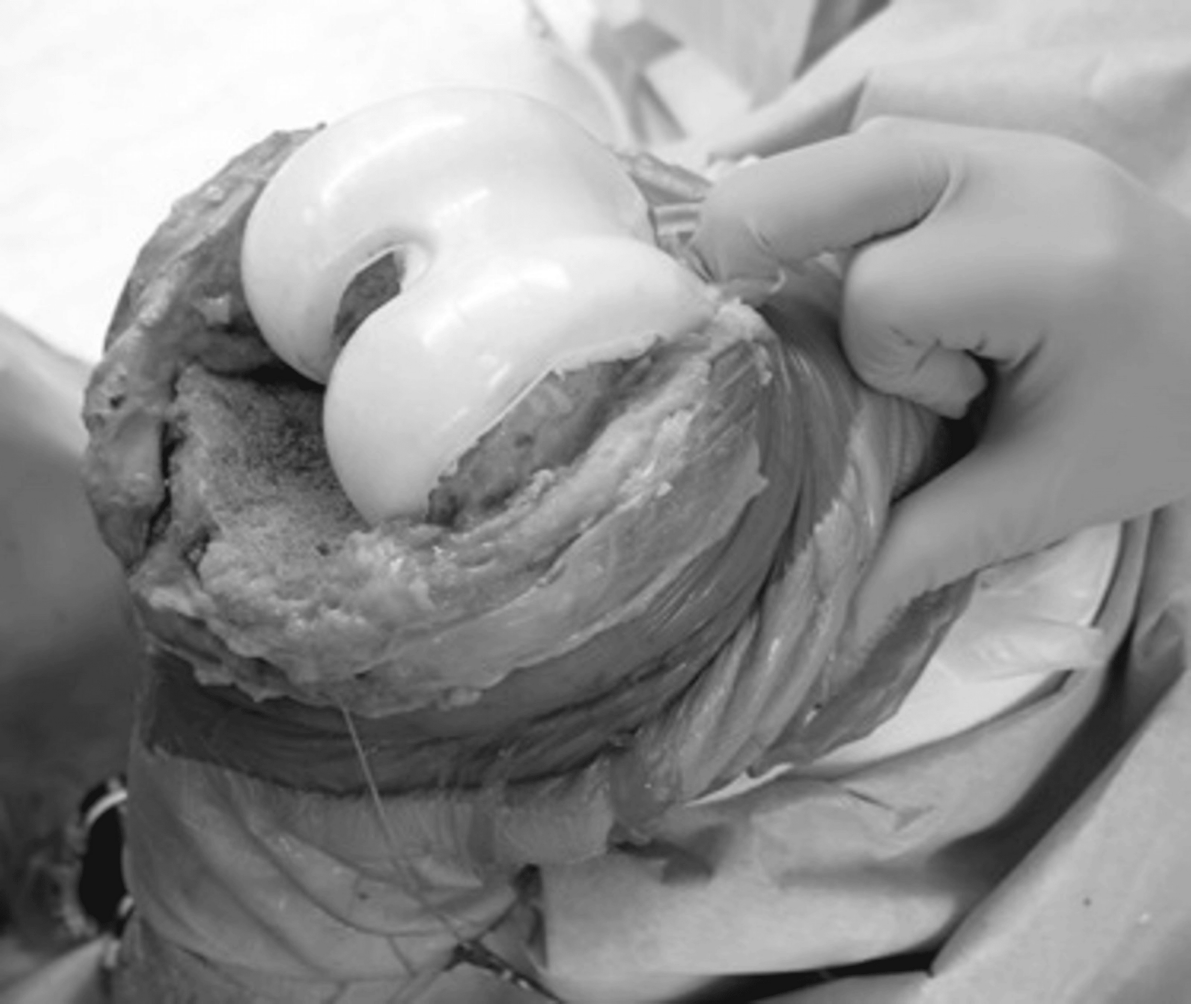
A photograph of the femoral mold being placed directly on the femur.

**Figure 3:**
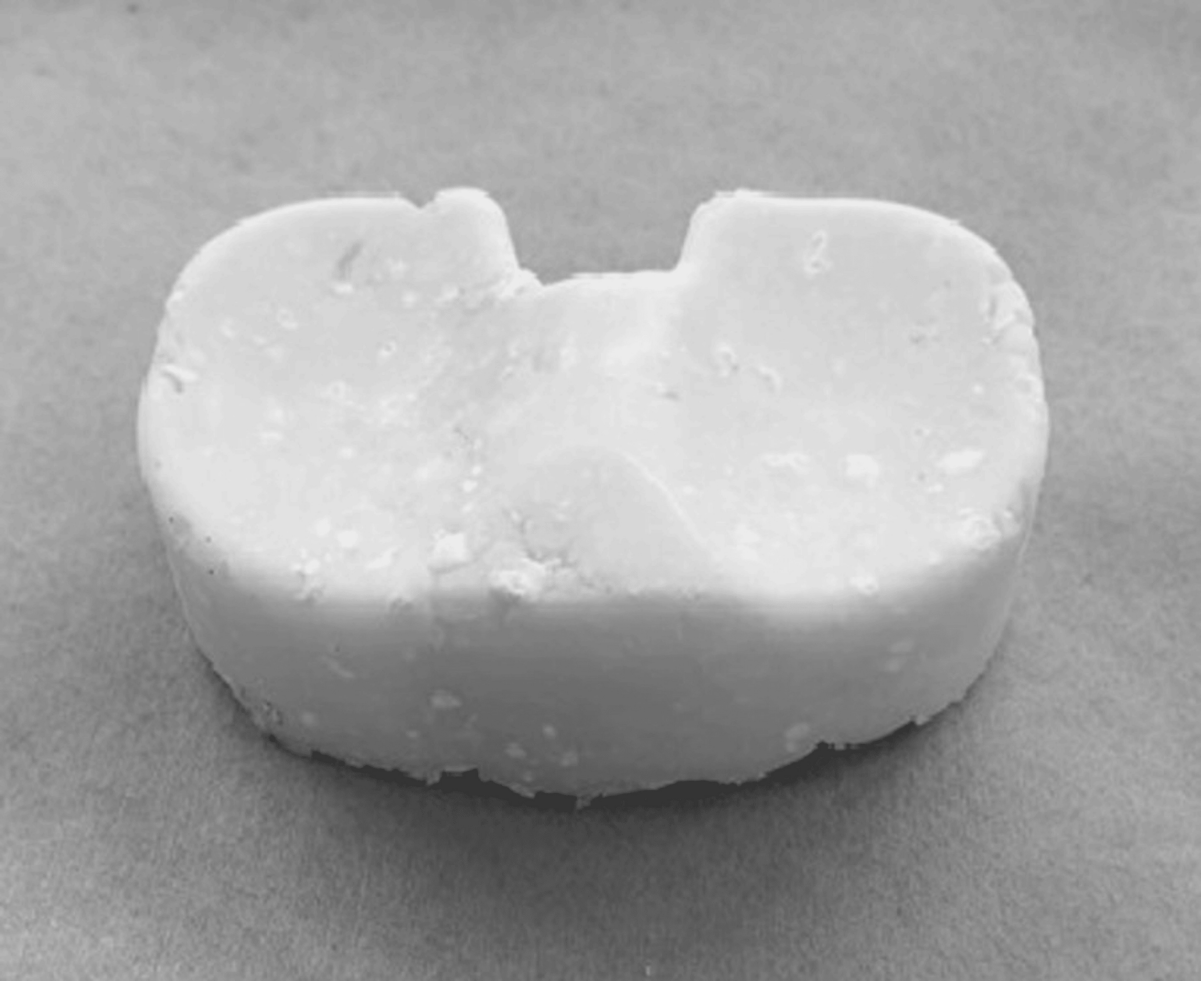
A photograph of the tibia side spacer, which was harden in advance with thickness adjusted according to the gap space.

**Figure 4:**
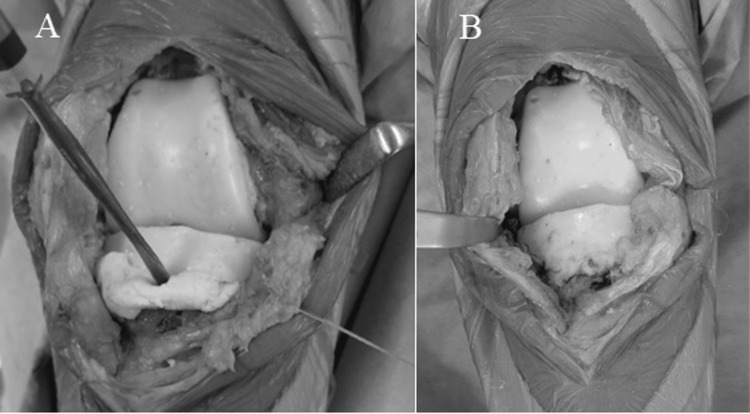
A photograph of the tibial side cement spacer insertion technique Additional cement containing antibiotics was used below the tibial mold to fill the gap space at the knee extension position (A). The appearance after the cement spacer was placed, leaving minimal joint gap, is shown in (B).

In all cases, the surgery was performed with a tourniquet, with the patient in the supine position. All components of the prosthetic, cement, surrounding fragile soft tissue, and bone were removed as far as possible through a medial parapatellar approach. In case of UKA infection, femoral and tibial osteotomy was required to insert the cement spacers. The appropriate size of the spacer molds (Figure 1) was determined based on the removed components and the shape of the osteotomy site on the femoral and tibial sides. At least three 40 g bags of cement (Simplex P Bone Cement; Stryker, Limerick, Ireland), each containing 2.0 g of vancomycin, were prepared to restore the bone defect, minimize dead space, and maintain a functional joint. First, before the cement was fully cured (late dough phase), the mold was directly placed on the femur (Figure 2). Subsequently, the mold for the tibia side was hardened in advance (Figure 3), and cement was added below the space of the tibial mold to attain joint gap balance (Figure 4). After suture closure of the bursa with monofilament, 2.0 g tranexamic acid and 40 mg gentamicin were injected without leakage.

Postoperative care between the stages

Postoperatively, wheelchair transfer training was initiated early. Weight-bearing was restricted for the first week to allow for wound rest. After that, all patients were allowed to walk with unrestricted weight-bearing gait with knee extension support. 

Based on the causative organism and antibiotic sensitivity results, intravenous antibiotics were used for at least four weeks, followed by a minimum of two weeks of oral antibiotics. If no causative organism was detected, antibiotics were selected empirically. This regimen continued until the following conditions were met: the patients' body temperature returned to normal (< 37.0°C), local symptoms and signs had disappeared, and a progressive decline in C-reactive protein (CRP) levels was observed. Antibiotic therapy was discontinued once these criteria were satisfied, based on reports that implementing a drug holiday before reimplantation has a positive impact on the mid-term outcomes of PJI treatment [13]. For patients who did not meet these criteria, oral antibiotic therapy was continued without interruption until reimplantation surgery. Joint aspiration was performed in cases of joint effusion or a significant increase in CRP. In patients with persistently elevated CRP levels but without clinical signs of infection, including joint fluid analysis, and those requiring prolonged cement retention, reimplantation surgery was undertaken with consideration for surgical modifications based on intraoperative findings. If the synovial fluid analysis revealed an elevated cell count or bacterial presence, it was considered a recurrence of infection, and either debridement or replacement of the cement spacer was performed.

Surgical technique for second-stage of revision surgery (reimplantation)

In all cases, an air tourniquet was applied to the femur to minimize intraoperative bleeding. The femoral and tibial cement spacers were meticulously removed. Bone resections were performed using implant guides to achieve a mechanical alignment of 90° to the mechanical axis in the frontal plane for both the femur and tibia. Depending on the extent of bone defects, augments and long stems were employed, and the implants were securely fixed using bone cement. After suturing the bursa with monofilament, 2.0 g of tranexamic acid and 40 mg of gentamicin were injected.

Postoperative management after reimplantation 

Postoperatively, the antibiotics that were effective during the first stage were administered intravenously for one week, followed by oral antibiotics for an additional three weeks. Range-of-motion and gait training were initiated in the early postoperative period. In cases of severe localized swelling with blister formation or delayed wound healing, a brief period of rest lasting several days was required; however, no additional specific restrictions were imposed.

Evaluation parameters

Walking ability after cement spacer insertion during the interval between stages was assessed. We considered full weight-bearing achieved if patients could walk unaided, or if they were able to maintain a standing position without assistance, even if they used a cane to prevent falls. Conversely, if walking remained unstable even with a cane, if a walker was required, or if walking was not possible, weight-bearing was classified as partial.

Further, the following items were evaluated in both cement spacer insertion and reimplantation surgeries: occurrence of mechanical complications such as cement spacer or implant fractures, dislocations, loosening, and bone fractures; occurrence of postoperative infections; surgical time; and hidden blood loss (HBL), which was indirectly estimates using formulas based on anthropometric data and laboratory parameters [14,15]. HBL is an indicator used not only to assess intraoperative blood loss but also to evaluate the total blood loss during the perioperative period.

Knee Society Knee Score (KSS), Knee Society Function Score, and knee range of motion (ROM) were evaluated in patients who underwent reimplantation and were followed up for more than six months postoperatively.

## Results

All 16 patients were able to achieve full weight-bearing gait during the interval between the first and second stages. Out of 16 patients, one (6.3%) patient (female, 50.6 kg, and BMI 22.3) experienced the complication of cement spacer dislocation due to instability, but no weight-bearing-related complications were observed in other patients. The operative time for the first stage of cement insertion was 106.3 min (range, 62-184 min) and the amount of HBL was 309.8 mL (range, 21.2-1106 mL).

During the interval between the stages, infection was observed in one of 16 (6.3%) patients at 11 days after the cement spacer insertion (Case 4: Table 2). In that patient, cement spacer replacement was not performed. Instead, debridement of the surrounding tissue was carried out, and reimplantation surgery was performed 80 days later. The mean interval between first and second stages of intervention was 92.7 days (range, 55-130 days).

**Table 2:** Patient characteristics and outcomes in two-stage revision for knee prosthetic joint infection M: male, F: female, BMI: body mass index, ROM: range of motion, KSS: Knee Society Score, KS function score: Knee Society function score, MRSA: methicillin-resistant Staphylococcus aureus, CNS: coagulase negative Staphylococcus, MSSA: methicillin-sensitive Staphylococcus aureus, M. intracell: Mycobacterium intracellulare, S. capitis: Staphylococcus capitis, S. epidemidis: Staphylococcus epidermidis † Follow-up after reimplantation ‡ average flex ROM after reimplantation

Case no.	Age (year)	Sex	BMI (kg/m^2^)	Onset of postoperative infection (weeks)	Organism	Interval between stages (days)	Complication during the waiting period	Complication after reimplantation	Follow-up (days)^†^	Latest outcome		
										Flex ROM^‡^ (degree)	KSS	KS function score
1	77	F	25.7	251.4	MRSA	121	-	Infection	-	-	-	-
2	82	M	22.6	137.6	MRSA	166	-	Infection	-	-	-	-
3	58	M	23.7	149.3	CNS	87	-	-	879	100	85	65
4	94	F	22.3	452.6	Negative	-	Spacer dislocation	-	-	-	-	-
				-	Negative	101	-	-	1103	120	89	45
5	59	M	33.4	27	MSSA	109	-	-	861	100	80	75
6	70	M	25.5	539	MSSA	130	-	-	641	110	82	90
7	73	M	29.9	2.7	MSSA	100	-	-	449	130	91	70
8	82	F	24.3	10.6	MSSA	91	-	-	450	100	95	95
9	83	F	23.4	20.1	MSSA	55	-	-	623	95	84	55
10	78	M	25.5	10.9	MSSA	84	-	-	357	130	81	100
11	71	M	24	29.9	MSSA	72	-	-	153	125	100	70
12	72	F	19.3	3.9	MRSA	91	Infection	-	272	110	92	80
13	70	F	25.1	19.4	CNS, Candida glabrata	114	-	-	187	100	62	55
14	69	F	23.2	206	M.intracell	80	-	-	191	110	90	65
15	63	F	34.9	18	S.capitis, S.epidermidis	85	-	-	197	125	90	90
16	68	F	23.1	23.3	MSSA	99	-	-	117	110	88	75
Mean ± SD	73.1 ± 9.4	-	25.4 ± 4.1	108.0 ± 175.4	-	92.7 ± 18.5	-	-	871 ± 188.8	111.8 ± 11.7	86.4 ± 8.6	73.6 ± 15.6

Before reimplantation, antibiotic use was stopped preoperatively for 48.1 days (range, 7-112 days) in nine patients, whereas oral antibiotics were continued in seven patients. The mean final CRP level before reimplantation was 0.7 mg/dL (range, 0.07-2.62 mg/dL). 

In two out of 16 (12.5%) patients, infection occurred after reimplantation (Cases 1 and 2: Table 2). In these cases, the implant was removed, and cement spacer insertion was performed. In the other 14 patients who underwent successful reimplantation after the first stage surgery of cement spacer insertion, the mean flexion ROM was 110.1° (range, 95°-130°), the mean KSS was 86.4 (range, 62-100), and the mean Knee Society Function Score was 73.6 (range, 45-100) at the latest follow-up. The mean follow-up period was 871 days (range, 117-1103 days) after reimplantation. The operative time for the second stage of reimplantation was 116.1 min (range, 76-153 min) and the amount of HBL was 476.1 mL (range, 89.5-859) for the second stage. The general clinical data are presented in Table 2.

## Discussion

Insall et al. [16] reported that a recommended interval between first and second stages of intervention of six weeks. However, this period may be further prolonged, which is sufficient to compromise knee joint function and reduce patient activity levels. Given this risk, strategies to optimize patient outcomes during this waiting period are critical. Elderly patients, in particular, are likely to manage better with full weight-bearing gait while awaiting reimplantation [12]. In our series, management with unrestricted weight-bearing and moderate ROM exercises might result in better postoperative outcomes in our series compared to the previously reported outcomes [3,9-12,17-22], despite the high average age and relatively short-term follow-up (Table 3).

**Table 3:** Recent studies of single / two-stage revision outcome for knee PJI PJI: prosthetic joint infection, Single: single stage revision, Two-stage: two-stage revision, KSS: Knee Society Score, ROM: range of motion, NR: not reported, A: articulating spacer, S: static spacer, FWB: full-weight bearing, PWB: partial-weight bearing, NWB: non-weight bearing

Study	Year	Number of knees	Age (years)	Follow-up (years)	Single/ Two-stage	Spacer (Articulating / Static)	Weight-bearing	Awaiting time for revision	KSS	Knee Society Function score	flex ROM (degree)	Mechanical Complication (%)	Infection (%)
Shen et al. [3]	2010	10	68.9	2.5	Two	A	PWB	7.8 months	83.6	75.5	96.7	2 (20%)	0%
Westrich et al. [9]	2010	75	72	4.4	Two	A	NWB	3.8 months	90.1	90	NR	NR	9.30%
Tian et al. [10]	2018	25	64.9	5.4	Two	A	NWB	11.5 weeks	83	78	94	5 (20%)	0
Van Thiel et al. [11]	2011	60	66	2.9	Two	A	39 (65%); PWB, 16 (26%); FWB 5 (8.3%); NWB	75 days	78.6	NR	101.3	1 (1.7%)	7 (12%)
Tsai et al. [12]	2019	32	70.4	3.1	Two	A	PWB	8.8 months	NR	NR	102	2 (6.3%)	4 (12.5%)
Emerson et al. [17]	2002	22 26	65.1 65.7	3.8 7.5	Two	A S	PWB	6 to 12 weeks	NR	NR	107.8 93.7	1 (3.8%)	2 (9.0%) 2 (7.6%)
Bauer et al. [18]	2006	30 77	71.8 68.3	4.5 4.5	Single Two	- Both	NR	NR	75.5 74.8	62.5 62.5	92.5 93	NR	33% 33%
Haddad et al. [19]	2015	28 74	63 68	2	Single Two	- A	- PWB	- 62days	88 76	NR	NR	NR	0 5 (7%)
Lichstein et al. [20]	2016	109	67	3.7	Two	S	NR	≧6 weeks	86	85	100	NR	6%
Current study	−	16	73.1	2.4	Two	A	FWB	92.7 days	86.4	73.6	110.1	1 (6.4%)	2 (12.5%)

Weight-bearing helps maintain flexibility [23], which is believed to influence both the operative time and postoperative range of motion. Blood loss and transfusion are risk factors for reinfection [24], but shorter operative time also contributes to a reduction in blood loss. The operative time for reimplantation in our series was 116.1 minutes (range, 76-153 minutes), which was shorter than the 141 ± 66.7 minutes reported in previous studies [25], and none of the patients required blood transfusions.

However, the spacer must be able to withstand weight bearing because an interim spacer exchange in two-stage revision is associated with worse patient outcomes [22]. Additionally, the stress from weight-bearing cannot be excluded as having a negative impact on local rest necessary for infection resolution. Thiel et al. [11] reported cement-on-cement spacers, in which a minimum of 4.0 g of antibiotics per package of cement was used; in this study, 16 patients were allowed to perform weight bearing as tolerated, and there was one (6.3%) spacer breakage in total. In the present study, even the patient who was the most overweight (male, 90.5 kg, and BMI 33.4) was able to walk with full weight-bearing gait during the 109-day interval until reimplantation without complication of cement spacer damage in the present study. In this study, we used at least three packs (120 g) of cement, which was sufficient to fill the bone defect and achieve a stable extension gap; further, the spacer had a high cement concentration (2.0 g of vancomycin per 40 g of cement), which may have contributed to the low incidence of cement spacer damage. It is recommended that the spacer contain 3.6 g of antibiotics for 40 g of cement [2,25], while Nodzo et al. [26] reported that there was no relationship between raw quantities of vancomycin and aminoglycoside in the spacer and successful outcomes. One of the complications we encountered during weight-bearing management was dislocation of the cement spacer. This dislocation resulted from a technical error, specifically the insufficient filling of the intra-articular gap, which suggests that appropriate measures could improve and prevent such occurrences.

The present study has several limitations. First, this study has a small sample size and the lack of a control group. Second, as this study only includes patients who completed the two-stage revision, selection bias may exist in the data regarding treatment outcomes and complication rates with unrestricted weight-bearing. Therefore, it is not yet clear whether performing weight-bearing actually contributed to improved postoperative outcomes. Further randomized controlled trials with a larger cohort are needed to support the findings presented here. Third, it was a limited evaluation of full weight-bearing gait in a group of patients with low body weight compared to that of Caucasians. Fourth, the protocol of antibiotic treatment before and after the surgery was not strict. The type of antibiotics, duration of use, and antibiotic-free periods were adjusted for each patient based on their physical findings and blood test results, including CRP level, white blood cell count, and renal function results. Fifth, the follow-up period at the final observation was relatively short, so the risk of developing a late infection was low, and the actual infection rate may have been underestimated.

## Conclusions

Management with unrestricted weight-bearing gait using cement spacers is a useful treatment option during the waiting period for two-stage revision surgery for knee PJI, because the postoperative outcomes after reimplantation were favorable even with a relatively short follow-up period. However, since complications related to weight-bearing may arise, the placement of stable cement spacers is essential. Further studies are needed to confirm whether the benefits of performing weight-bearing outweigh the risk of complications and lead to improved postoperative outcomes.
